# Acute morphine alters GABAergic transmission in the central amygdala during naloxone-precipitated morphine withdrawal: role of cyclic AMP

**DOI:** 10.3389/fnint.2014.00045

**Published:** 2014-06-04

**Authors:** Michal Bajo, Samuel G. Madamba, Marisa Roberto, George R. Siggins

**Affiliations:** ^1^Committee on the Neurobiology of Addictive Disorders, The Scripps Research InstituteLa Jolla, CA, USA; ^2^Department of Molecular and Cellular Neuroscience, The Scripps Research InstituteLa Jolla, CA, USA

**Keywords:** opioids, dependence, GABA, G-protein, cAMP, withdrawal, morphine

## Abstract

The central amygdala (CeA) plays an important role in opioid addiction. Therefore, we examined the effects of naloxone-precipitated morphine withdrawal (WD) on GABAergic transmission in rat CeA neurons using whole-cell recordings with naloxone in the bath. The basal frequency of miniature inhibitory postsynaptic currents (mIPSCs) increased in CeA neurons from WD compared to placebo rats. Acute morphine (10 μ M) had mixed effects (≥20% change from baseline) on mIPSCs in placebo and WD rats. In most CeA neurons (64%) from placebo rats, morphine significantly decreased mIPSC frequency and amplitude. In 32% of placebo neurons, morphine significantly increased mIPSC amplitudes but had no effect on mIPSC frequency. In WD rats, acute morphine significantly increased mIPSC frequency but had no effect on mIPSC amplitude in 41% of CeA neurons. In 45% of cells, acute morphine significantly decreased mIPSC frequency and amplitude. Pre-treatment with the cyclic AMP inhibitor (R)-adenosine, cyclic 3',5'-(hydrogenphosphorothioate) triethylammonium (RP), prevented acute morphine-induced potentiation of mIPSCs. Pre-treatment of slices with the Gi/o G-protein subunit inhibitor pertussis toxin (PTX) did not prevent the acute morphine-induced enhancement or inhibition of mIPSCs. PTX and RP decreased basal mIPSC frequencies and amplitudes only in WD rats. The results suggest that inhibition of GABAergic transmission in the CeA by acute morphine is mediated by PTX-insensitive mechanisms, although PTX-sensitive mechanisms cannot be ruled out for non-morphine responsive cells; by contrast, potentiation of GABAergic transmission is mediated by activated cAMP signaling that also mediates the increased basal GABAergic transmission in WD rats. Our data indicate that during the acute phase of WD, the CeA opioid and GABAergic systems undergo neuroadaptative changes conditioned by a previous chronic morphine exposure and dependence.

## Introduction

The central nucleus of the amygdala (CeA) is a part of the neuroanatomical entity termed the extended amygdala that also includes the bed nucleus of the stria terminalis (BNST), and a transition zone in the medial (shell) subregion of the nucleus accumbens (Heimer and Alheid, 1991). The extended amygdala is considered a common anatomical substrate integrating brain arousal–stress systems with hedonic processing systems producing the emotional states that promote negative reinforcement mechanisms associated with the development of addiction (Koob and Volkow, 2010). In addition, the extended amygdala has long been hypothesized to play a key role in the emotional component of pain processing (Neugebauer et al., 2004), and in the mediation of the affective signs of withdrawal from acute morphine treatment (Criner et al., 2007) and the motivational aspects of chronic opiate withdrawal (Frenois et al., 2002). The CeA has a key function in the acute reinforcing actions of drugs of abuse, including opioids (Koob and Volkow, 2010), and is considered to be critical for the affective component of acute and chronic opiate withdrawal (Jin et al., 2004).

Our recent study and those from others have shown that not all CeA neurons are responsive to acute opioids, suggesting the presence of neuronal subpopulations mediating the opioid effects in the CeA (Zhu and Pan, 2004, 2005; Finnegan et al., 2005, 2006; Chieng et al., 2006; Bajo et al., 2011). Most CeA neurons in rodents are GABAergic neurons with inhibitory recurrent or feed-forward connections, as well as inhibitory projections to brainstem (Sun and Cassell, 1993; Cassell et al., 1999) and other extended amygdala areas such as BNST (Weller and Smith, 1982; Veinante and Freund-Mercier, 1998). Activation of opioid receptors is reported to induce inhibitory potassium currents in subpopulations of CeA neurons characterized by expression of mu (MORs) and kappa (KORs) opioid receptors (Zhu and Pan, 2004; Chieng et al., 2006).

MORs tonically inhibit GABAergic transmission in the CeA and acute activation of MORs diminishes GABAergic transmission in most CeA neurons, predominantly by reducing presynaptic GABA release (Kang-Park et al., 2009; Bajo et al., 2011). Moreover, in the CeA neurons projecting to periaqueductal gray area (PAG), presynaptic MORs primarily attenuate GABAergic synaptic transmission (Finnegan et al., 2005). In the central extended amygdala chronic morphine induces transcriptional plasticity characterized by a dysregulation of expression of genes involved in neurogenesis, cell growth and signaling (Bajo et al., 2006; Befort et al., 2008). Heroin self-administration alters gene expression of opioids in the CeA (Solecki et al., 2009). In amygdala, chronic heroin administration decreases opioid agonist-stimulated GTP binding but does not alter MOR binding autoradiography (Sim-Selley et al., 2000; Maher et al., 2005). In contrast, no changes in the GTP binding have been observed in amygdala after chronic morphine (Sim et al., 1996) or in the CeA during morphine withdrawal (Kirschke et al., 2002). The ERK signaling pathway in the CeA is critical in induction of morphine craving (Li et al., 2008). We previously found no significant effect on basal GABAergic transmission during chronic morphine treatment, suggesting a lack of effect of long-term morphine, or tolerance and alterations of MOR-dependent postsynaptic mechanisms (Bajo et al., 2011).

Repeated morphine-conditioning treatment enhances glutamate synaptic strength and recruits new functional DORs on glutamate synapses in the CeA (Sim et al., 1996; Sim-Selley et al., 2000; Kirschke et al., 2002; Maher et al., 2005; Befort et al., 2008; Li et al., 2008; Bie et al., 2009; Solecki et al., 2009). These findings indicate that chronic opioid exposure followed by subsequent withdrawal induces complex neuroadaptation of the opioid and other systems in the CeA. The mechanisms mediating such adaptive changes likely involve both pre- and postsynaptic mechanisms and lead to alteration of MOR-dependent modulation of the physiological and synaptic properties of the amygdala neurons.

In a previous study (Bajo et al., 2011) characterizing adaptation of the GABAergic system during continuous chronic morphine exposure we found a tolerance to inhibitory effects of acute activation of MORs on presynaptic GABA release in the CeA. Moreover, we found very similar effects of acute application of MOR agonist and antagonist on GABAergic transmission in placebo- and morphine-treated rats, further suggesting a homeostatic neuroadaptation of the CeA opioid system and MOR-dependent regulation of CeA GABAergic transmission. Interestingly, we observed significant effects of acute MOR agonist on the mIPSC amplitudes only in CeA of morphine-treated rats, but not of placebo rats, suggesting postsynaptic adaptative changes of the MOR system during chronic morphine treatment (Bajo et al., 2011). Therefore, in the present study, we aimed to assess the changes of the MOR-dependent modulation of GABAergic transmission associated with an early phase of morphine withdrawal. We hypothesized that, as in other brain regions (Christie, 2008), the CeA opioid system will be over-activated during morphine withdrawal. Therefore, here we have examined basal CeA GABAergic transmission during morphine withdrawal and challenged the MOR system by acute morphine application to examine the influence of activated MORs on GABAergic transmission at a withdrawal time-point. To model naloxone-precipitated morphine withdrawal, we performed all recordings with naloxone (placebo rats) and naloxone plus morphine co-application (morphine WD rats) in the bath solution. In the ventral tegmental area Madhavan et al. (2010a) found an acute morphine-induced facilitation of GABAergic transmission, indicating a switch in the coupling of MORs from inhibitory Gi/o subunit to stimulatory Gs subunit signaling. These findings are in accord with our preliminary report on the facilitatory effects of acute morphine application on CeA GABAergic transmission during spontaneous morphine withdrawal (Bajo et al., 2008, 2009). Thus, we hypothesized that adaptative changes of MOR and GABAergic systems similar to those reported by Madhavan et al. are present in CeA during naloxone-precipitated morphine withdrawal.

Our results show alterations of the basal GABAergic transmission in morphine WD rats, and acute morphine-induced increases and decreases of CeA GABAergic transmission in both placebo and WD rats. Furthermore, as Gi/o are the primary G subunits coupled to MORs (Laugwitz et al., 1993; Clark et al., 2006), we hypothesized that inhibitory effects of acute morphine application on CeA GABAergic transmission are mediated by Gi/o, whereas the facilitatory effects of acute morphine involve Gs subunits and activation of cAMP-PKA signaling. To test this hypothesis, we applied pertussis toxin (PTX), an inhibitor of Gi/o subunits, and (R)-adenosine, cyclic 3',5'-(hydrogenphosphorothioate) triethylammonium (RP), an inhibitor of the cAMP-PKA pathway. We found that PTX-insensitive mechanisms are involved in the acute morphine-induced inhibition of GABAergic transmission, but we cannot completely rule out an involvement of PTX-sensitive mechanisms in the inhibitory effects of acute morphine. However, activated cAMP signaling appears to mediate the morphine withdrawal-elicited increase of basal GABAergic transmission and the acute morphine-induced facilitation of GABAergic transmission in CeA.

## Materials and methods

### Animal treatment

Male Sprague-Dawley rats (weights at the time of electrophysiological recordings: placebo: 218 ± 8 g; *n* = 25; morphine: 216 ± 11 g; *n* = 24) were housed in a temperature- and humidity-controlled room on a 12-h light/dark cycle (lights on at 6:00 am) with food and water available *ad libitum*. We used the standard morphine pellet method, with two morphine (75 mg of morphine/pellet) or placebo pellets implanted subcutaneously (Gold et al., 1994; Bajo et al., 2011). To precipitate morphine withdrawal, we injected naloxone (1 mg/kg) subcutaneously. Just prior to sacrifice, the rats were then observed for development of physical signs of morphine withdrawal, represented by diarrhea and wet dog shakes. These signs developed solely in chronic morphine-treated rats within 15–20 min after the naloxone injection. We conducted all care and surgical procedures in accordance with the National Institutes of Health Guide for the Care and Use of Laboratory Animals and with the Institutional Animal Care and Use Committee (IACUC) policies of The Scripps Research Institute.

### Slice preparation

Rats were anesthetized with 3% isoflurane, decapitated and the brains quickly removed and placed in ice-cold oxygenated artificial cerebrospinal fluid (ACSF; in mM: 130 NaCl, 3.5 KCl, 1.25 NaH_2_PO_4_.H_2_O, 1.5 MgSO_4_.7H_2_O, 2.0 CaCl_2_.2H_2_O, 24 NaHCO_3_, and 10 glucose) gassed with 95% O_2_ and 5% CO_2_. We cut coronal slices (300–400 μ M) containing the CeA using a Leica 1000S vibrotome cutter (Campden, Lafayette, Indiana). To simulate *in vivo* conditions of naloxone-induced withdrawal during our recordings in morphine dependent rats, we included naloxone (10 μ M) and morphine (1 μ M) in the cutting solution during slice preparation and in the bath during recordings. The slice preparation and recordings from placebo (morphine naïve) rats were conducted in the ACSF containing naloxone (10 μ M). We performed recordings within 1–6 h after slice cutting. Drugs were added to the ACSF from stock solutions (flow rate 2–4 ml/min) in known concentrations and we took all physiological measures before acute morphine (baseline) and during its superfusion (5–15 min). To avoid multiple morphine applications, we recorded from a single neuron from each slice and discarded all other slices exposed to acute morphine.

### Whole-cell patch-clamp recording

We performed whole-cell patch-clamp recording in voltage clamp mode as described previously (Bajo et al., 2011). To facilitate cell identification, we used infrared/DIC visualization, followed by digitization and image enhancement, via an upright, fixed-stage Olympus microscope. We used a 40X water immersion lens and image processing with EXI Blue CCD camera (QImaging software, Surrey, BC, Canada). We isolated miniature GABA_A_ IPSCs (mIPSCs) pharmacologically by applying 20 μ M DNQX, 30 μ M DL-AP5, and 1 μ M CGP 52432 and 1 μ M tetrodotoxin (TTX) to the bath. To evaluate a role for Gi/o and cAMP, we pre-treated CeA slices with 250 ng/ml of PTX for 0.5 up to 6 h, an inhibitor of Gi/o subunits, and 500 nM (R)-adenosine, cyclic 3',5'-(hydrogenphosphorothioate) triethylammonium (RP), an inhibitor of cAMP signaling, for 15 min prior to and throughout the recordings. All mIPSC recordings were held at—60 mV membrane potential and were made with 3–4 MΩ pipettes filled with an internal solution containing (in mM): 135 KCl, 10 HEPES, 2 MgCl_2_, 0.5 EGTA, 5 ATP, and 1 GTP (the latter two added fresh on the day of recording), pH 7.3–7.4, osmolarity 275–290 mOsm. For data acqusition, we used a Multiclamp 700B (Molecular Devices) and pClamp 10.2 software (Molecular Devices).

### Data analysis and statistics

To analyze data acquired from whole-cell recordings, we used Clampfit 10.2 (Molecular Devices) and MiniAnalysis 5.1 software (Synaptosoft, Leonia, NJ), respectively. We used GraphPad Prism 5.0 software (GraphPad Software, San Diego, CA) for all statistical analyses of results obtained by intracellular and whole-cell recordings. Because not all CeA neurons are responsive to MOR agonists (Zhu and Pan, 2004; Chieng et al., 2006), we used a change of 20% of control values as a threshold for dividing the cells into MOR agonist-sensitive and -insensitive groups (Bajo et al., 2011). We accepted statistical significance at the *p* < 0.05 level using One- and Two-Way ANOVA, the Kolmogorov-Smirnoff test, and *t*-tests.

### Drugs

We purchased CGP 55845A, DNQX, DL-AP5, and PTX from Tocris Biosciences (Ellisville, MI), TTX from Calbiochem (San Diego, CA) and RP from Sigma-Aldrich (St. Luis, MO). Morphine sulphate, morphine, and placebo pellets were provided by the National Institute on Drug Abuse.

## Results

### Effects of morphine and opiate withdrawal

**Figure 1:**
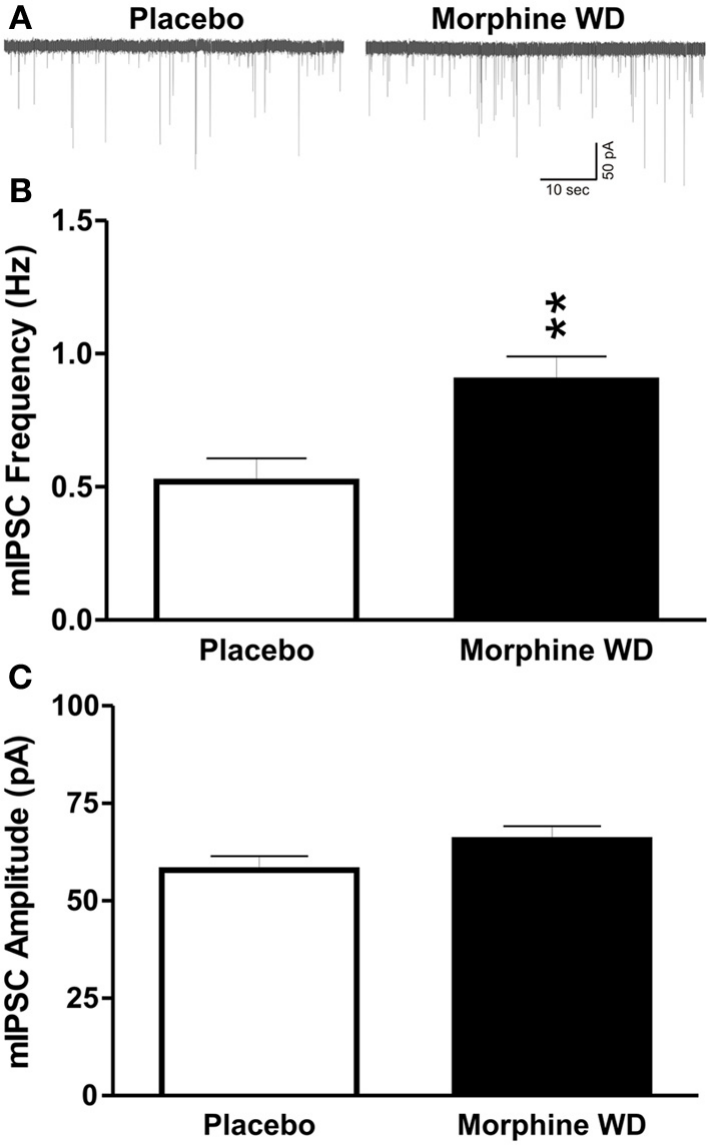
**Naloxone-precipitated withdrawal increases GABA release in the CeA from morphine-dependent rats. (A)** Representative GABA_A_ mIPSC recordings (in the presence of TTX) from CeA neurons from placebo (left column) and morphine-withdrawn rats (right column), respectively. **(B)** The mean basal mIPSC frequencies in CeA neurons (*n* = 29) from morphine-withdrawn rats were significantly increased (*p* < 0.01) compared to neurons (*n* = 25) from placebo (morphine naïve) rats. **(C)** There was no significant difference in the mean basal mIPSC amplitude between CeA neurons from placebo and morphine withdrawn rats. The statistical significance ^**^p < 0.01.

We recorded miniature IPSCs from 76 CeA neurons using whole-cell recordings in voltage-clamp mode. The basal frequencies of the CeA mIPSCs were significantly [*t*_(52)_ = 3.1, *p* < 0.01] increased in the morphine withdrawn (WD) rats (0.9 ± 0.1 Hz, *n* = 29) compared to the placebo rats (0.5 ± 0.1 Hz, *n* = 25), suggesting an increase in presynaptic GABA release during naloxone-precipitated WD. There was no significant difference in the mean mIPSC amplitudes between the CeAs of placebo (57.9 ± 3.6 pA, *n* = 25) and morphine WD rats [65.6 ± 3.5 pA, *n* = 29; *t*_(52)_ = 1.8, *p* > 0.05; Figure 1].

**Figure 2:**
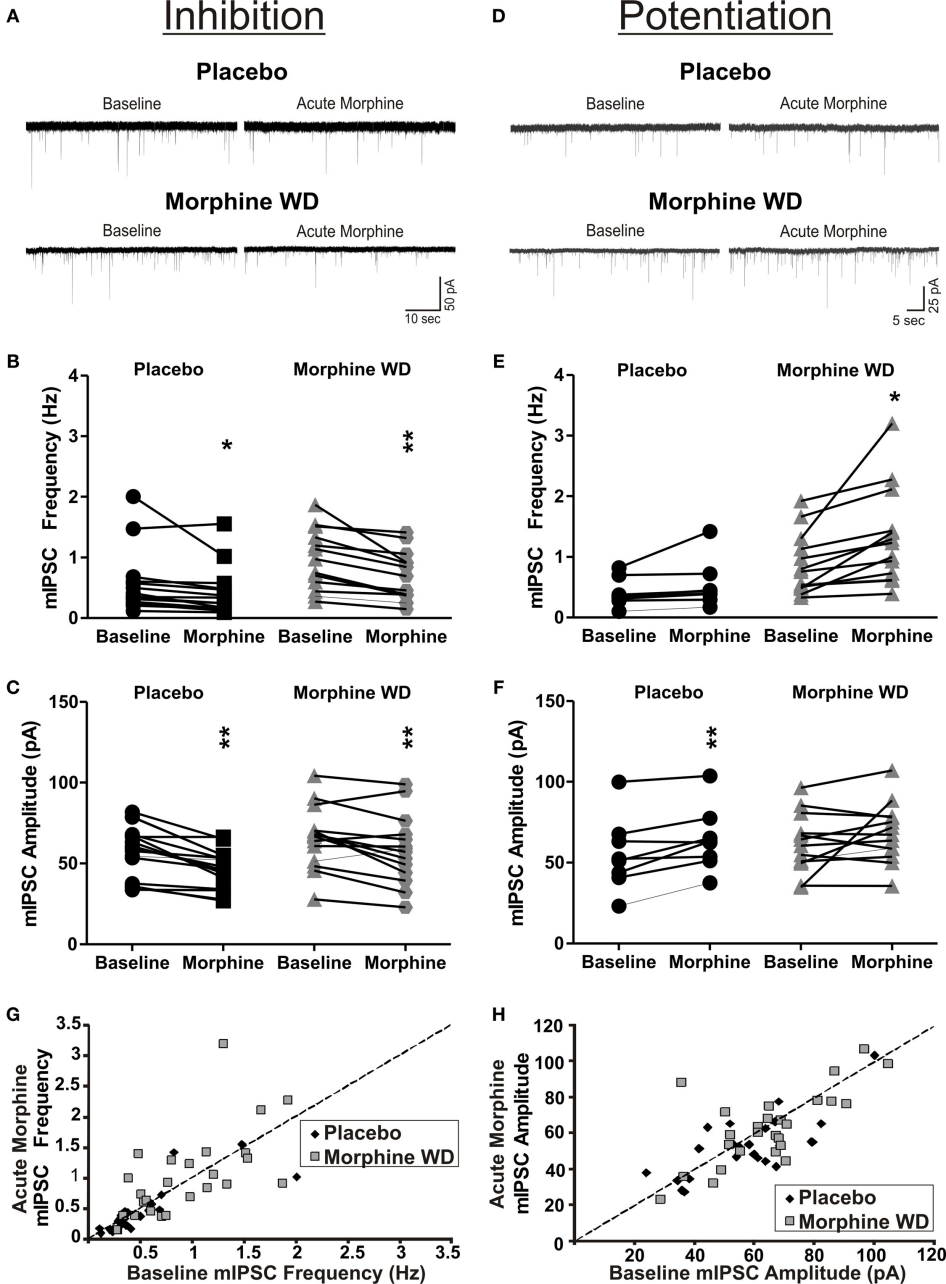
**Acute morphine has mixed effects on GABAergic transmission in the CeA following naloxone-precipitated withdrawal**. **(A)** Representative recordings from the CeA neurons responding to acute morphine (10 μ M) application by an inhibition of mISPCs from placebo (top panel) and morphine withdrawn (bottom panel) rats. **(B,C)** In 64% of neurons from placebo and 45% from morphine-withdrawn rats, acute morphine decreased the frequency and amplitude of the mIPSCs. **(D)** Representative recordings of CeA neurons from placebo and morphine-withdrawn rats that responded to acute morphine by an increase in the mIPSCs. **(E,F)** Whereas 32% of CeA neurons from placebo rats responded to acute morphine by an increase in mIPSC amplitudes, 41% of neurons from morphine-withdrawn rats responded to acute morphine by an increase in the mIPSC frequencies. **(G,H)** 2-D scatter graphs showing distribution of baseline (x-axis) and acute morphine (y-axis) mIPSC frequencies **(G)** and amplitudes **(H)** of individual acute morphine-responsive CeA neurons. The statistical significance ^*^ and ^**^ were set at *p* < 0.05 and at *p* < 0.01, respectively.

Acute application of morphine (10 μ M) produced mixed effects on the mIPSC frequencies and amplitudes in both placebo and morphine WD rats. We used a 20% change in the frequencies and/or amplitudes of the mIPSCs as the selection criterion (Bajo et al., 2011) and divided the CeA neurons into three groups: no response, increase, or decrease in the mIPSC measures. In placebo rats, the majority of CeA neurons (16 of 25 neurons) responded to acute superfused morphine with a decrease in both frequency [73. 0 ± 4.3% of baseline; *t*_(15)_ = 2.4, *p* < 0.05] and amplitude [81.4 ± 2.9% of baseline; *t*_(15)_ = 5.4, *p* < 0.05] of the mIPSCs (Figures 2A–C,G,H). Another subpopulation (8 of 25 neurons) of placebo CeA neurons responded to acute morphine application with a significant increase in the amplitude [122.3 ± 7.8% of baseline, *n* = 8; *t*_(7)_ = 3.7, *p* < 0.05] but not frequency of mIPSCs [129.2 ± 9.0% of baseline, *t*_(7)_ = 1.8, *p* >0.05; Figures 2D–H]. In the morphine WD rats, acute morphine application decreased both frequency [71.0 ± 4.2% of baseline, *n* = 13; *t*_(12)_ = 4.2, *p* < 0.05] as well as amplitude [86.2 ± 3.8%, *t*_(12)_ = 3.3, *p* < 0.05] of the mIPSCs in 13 of 29 CeA neurons. Another subpopulation of the CeA neurons (12 of 29 cells) responded to acute morphine with a significant increase in the frequency [163.9 ± 18.6%, *n* = 12; *t*_(11)_ = 3.3, *p* < 0.01] but not the amplitude of mIPSCs [116.8 ± 13.3%, *t*_(11)_ = 1.3, *p* > 0.05; Figures 2D–H]. Two-Way ANOVA, comparing the enhancing effects of acute morphine on the mIPSC frequencies between CeA neurons from placebo and morphine pellet treated and WD rats, showed significant main effects of acute morphine application [*F*_(1, 18)_ = 10.8, *p* < 0.01] and pellet treatment [*F*_(1, 18)_ = 7.9, *p* < 0.01], and no significant interaction between acute morphine and pellet treatment [*F*_(1, 18)_ = 3.5, *p* = 0.08]. The ANOVA analysis also showed a significant main effect of acute morphine [*F*_(1, 18)_ = 5.9, *p* < 0.05] but no main effect of the pellet treatment [*F*_(1, 18)_ = 0.5, *p* > 0.05] nor acute morphine × pellet treatment interaction [*F*_(1, 18)_ = 0.1, *p* > 0.05] on the mIPSC amplitudes. The comparison of the inhibitory effects of acute morphine on the mIPSCs between placebo and morphine WD rats showed a significant main effect of acute morphine [*F*_(1, 26)_ = 21.9, *p* < 0.01] and pellet treatment [*F*_(1, 26)_ = 6.3, *p* < 0.05] on mIPSC frequencies but no interaction between acute morphine and pellet treatment [*F*_(1, 26)_ = 2.4, *p* > 0.05]. Similarly, we found a significant main effect of acute morphine [*F*_(1, 26)_ = 34.5, *p* < 0.01] and pellet treatment [*F*_(1, 26)_ = 5.1, *p* < 0.05] on mIPSC amplitudes associated with no interaction between acute morphine and pellet treatment [*F*_(1, 26)_ = 0.7, *p* > 0.05]. Acute morphine had no effect on either the frequency or amplitude in the remaining 1 cell from placebo or 5 cells from morphine WD rats (data not shown). These results suggest that regardless of their previous morphine exposure, some CeA neurons adapt to prolonged (<8 h) exposure to naloxone during an induction of naloxone-precipitated withdrawal, by reversing the inhibitory effects of the acute morphine on GABAergic transmission. The results are summarized in Table 1.

**Table 1:** **Summary of acute morphine-induced changes in mIPSCs in CeA neurons of placebo and morphine (WD) pellet–treated rats**.

**Pellet**	**Basal mIPSCs**		**Acute Morphine (10 μM)**	
**treatment**	**Frequency (Hz)**	**Amplitude (pA)**	**Frequency (Hz)**	**Amplitude (pA)**
Placebo	0.52 ± 0.09	57.9 ± 3.6	↓	↑ and ↓
WD	0.91 ± 0.09^*^	66.4 ± 3.4	↑ and ↓	↓

* Significant difference between placebo and WD rats;

↓, significant decrease; ↑, significant increase.

### Signaling pathways involved in morphine and withdrawal effects

**Figure 3:**
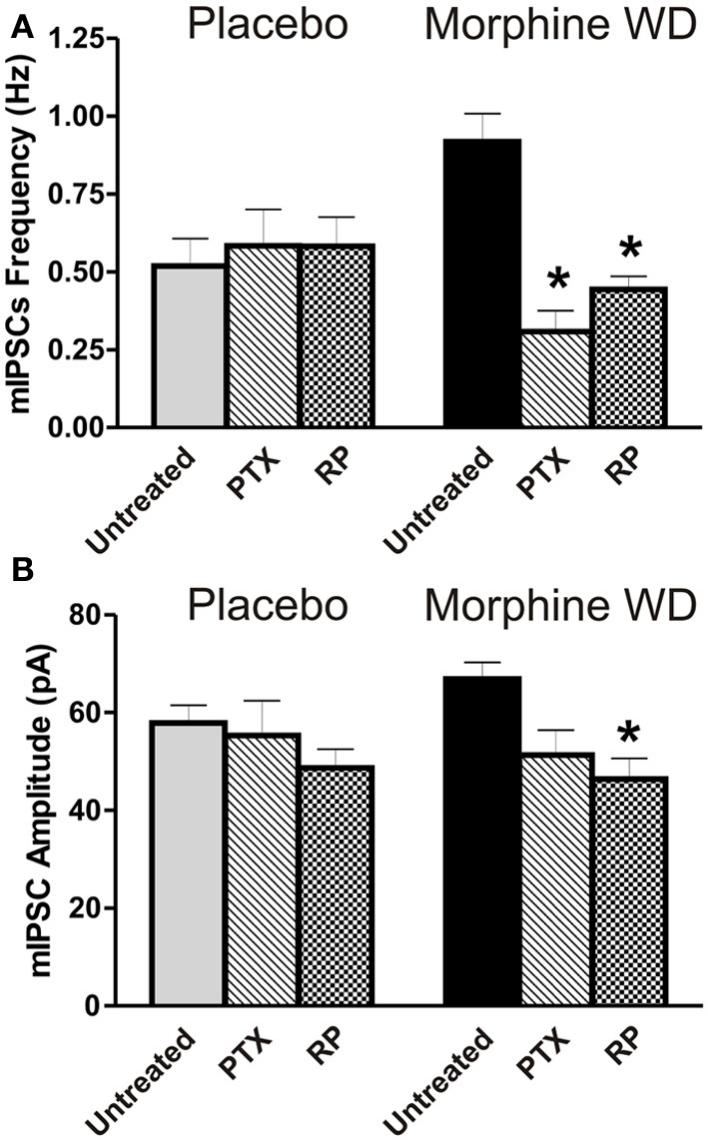
**Effects of Gi/o and cAMP inhibitors on CeA basal GABAergic transmission**. **(A)** The Gi/o inhibitor PTX (250 ng/ml) and cAMP inhibitor RP (500 nM) prevented an increase in mIPSC frequencies in CeA neurons (PTX: *n* = 6 and RP: *n* = 13) from morphine WD rats. In contrast, these agents had no effects on mIPSC frequencies in neurons (PTX: *n* = 9 and RP: *n* = 16) from placebo rats. The values presented as untreated CeA neurons correspond to those neurons shown in Figure 1A. **(B)** By comparison to the untreated CeA neurons (the same data presented in Figure 1B), RP significantly reduced mean basal mIPSC amplitudes only in neurons from morphine WD rats. In the presence of PTX, basal mIPSC amplitudes were not significantly different between placebo and morphine WD rats. Statistical significance: ^*^p < 0.05, was calculated by One-Way ANOVA.

We hypothesized that the observed mixed effects of acute morphine on GABAergic transmission in the CeA are caused by a difference in coupling of MORs to G protein subunits, resulting in activation instead of inhibition of the cyclic AMP (cAMP) pathway. To test this possible mechanism, we pre-incubated CeA slices with 250 ng/ml PTX, an inhibitor of Gi/o,or 500 nM RP, the cAMP signaling inhibitor. In CeA of placebo rats, the PTX (*n* = 9 cells from 4 rats) or RP (*n* = 16 cells from 4 rats) pretreatments had no significant effects on basal mIPSC frequencies [*F*_(2,49)_ = 0.2, *p* > 0.05] or amplitudes [*F*_(2,49)_ = 1.3, *p* > 0.05], compared to the untreated CeA neurons (Figure 3). These data suggest that PTX-sensitive Gi/o signaling and cAMP-PKA signaling are not critically involved in the basal GABAergic transmission in the CeA of the placebo rats during prolonged naloxone exposure.

In the morphine WD rats, there were significant differences in the basal mean frequency [*F*_(2, 47)_ = 10, *p* < 0.01] and amplitude [*F*_(2,36)_ = 12.3, *p* < 0.01] between PTX-, RP-pretreated and untreated CeA neurons. We found a significant reduction (Bonferoni test, *p* < 0.05) of the mIPSC frequencies in CeA neurons pretreated with PTX (0.3 ± 0.07 pA, *n* = 6 cells from 4 rats) or RP (0.4 ± 0.05 pA, *n* = 13 cells from 6 rats) compared to the untreated CeA cells (Figure 3). The Bonferroni's *post-hoc* test showed a significant decrease in the mean mIPSC amplitudes following RP (46.4 ± 4.2 pA) but not PTX pretreatment (51.3 ± 5.1 pA) compared to untreated CeA neurons (66.9 ± 3.4 pA, *n* = 29; *p* < 0.05). These findings suggest that both Gi/oand activated cAMP-PKA signaling pathways are involved in the modulation of increased presynaptic GABA release, whereas activated cAMP-PKA signaling may be involved also in the postsynaptic regulation of GABAergic transmission in the CeA during naloxone-precipitated withdrawal.

**Figure 4:**
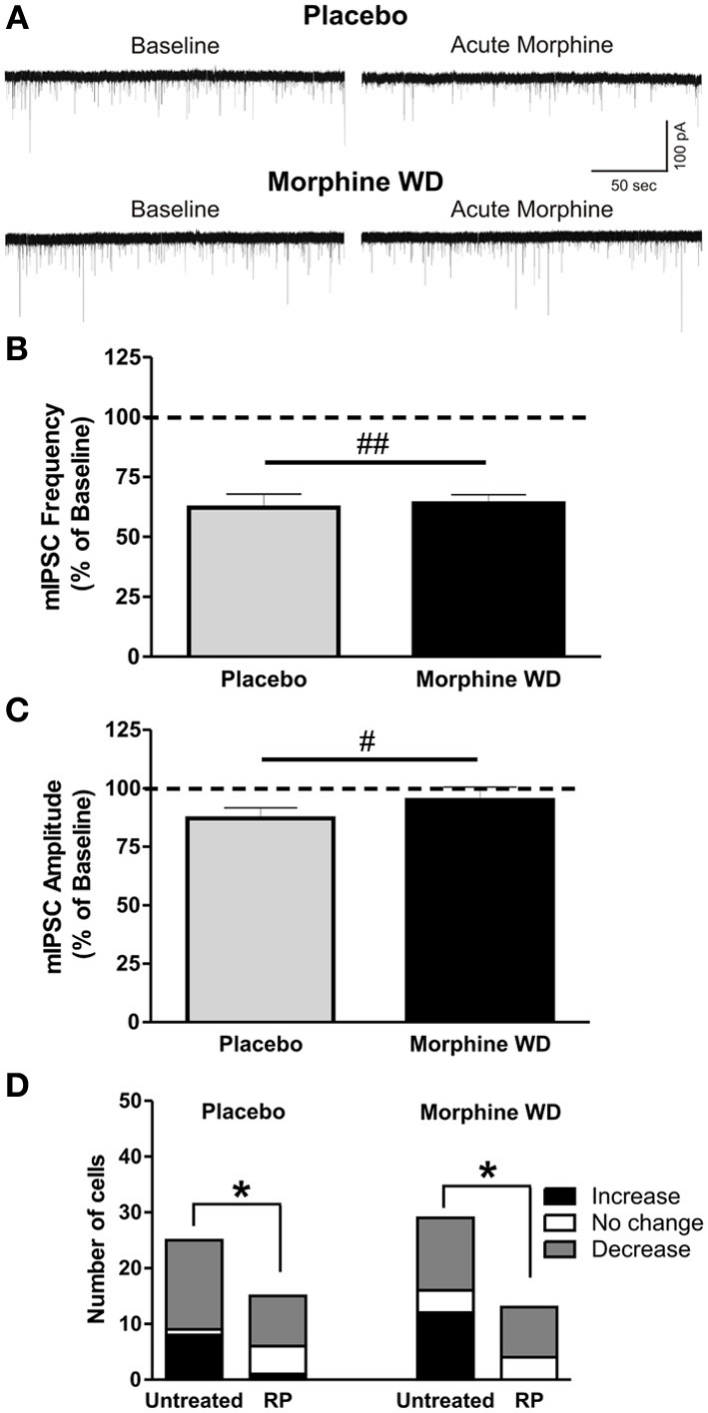
**RP inhibition of cAMP prevents enhancement of GABAergic transmission induced by acute morphine application**. **(A)** Represen- tative recordings from CeA neurons from placebo and morphine WD rats pre-treated with RP. **(B)** After RP (500 nM) pre-treatment, acute morphine (10 μ M) gave a significant main inhibitory effect on mean mIPSC frequencies and amplitudes **(C)** of CeA neurons from placebo (*n* = 10) and morphine-withdrawn (*n* = 9) rats. Statistical significance (^#^*p* < 0.05 and ^##^*p* < 0.01) was calculated by Two-Way ANOVA. **(D)** In CeA from both placebo and WD rats, RP-pretreatment significantly reduced the number of CeA neurons responding to acute morphine by an increase in mIPSCs, as calculated by Chi-square test (^*^p < 0.05).

After pre-incubation of CeA slices with RP for 15–30 min, acute morphine significantly decreased the mean mIPSC frequencies [*F*_(1, 37)_ = 16.23, *p* < 0.05] and amplitudes [*F*_(1, 37)_ = 5.8, *p* < 0.05; *t*_(10)_ = 12.5, *p* < 0.05] in 10 of 15 CeA neurons from 4 placebo rats (frequency: 62.2 ± 5.7% of baseline; amplitude: 87.1 ± 4.6% of baseline) and 9 of 15 neurons from 6 WD rats (frequency: 63.8 ± 3.8% of baseline; amplitude: 95.1 ± 5.7% of baseline; Figures 4A–C). There was no significant main effect of pellet treatment or interaction between acute morphine and pellet treatment. In placebo rats, there were no significant effects of acute morphine on the mIPSCs in 4 cells, and no potentiation of the mIPSC frequency and amplitude in one neuron. In the rest of the CeA neurons (4 of 13 cells) from WD rats, acute morphine had no significant effects on mIPSC frequency or amplitude. Thus, RP pre-incubation prevented the CeA neurons from responding to acute morphine with increases in mIPSCs in placebo (Chi-square test, *p* < 0.05) or WD (Chi-square test, *p* < 0.05) rats (Figure 4D). These results suggest that activation of the cAMP signaling pathway plays a key role in the acute morphine-induced increase in the CeA GABAergic transmission in a subpopulation of CeA neurons, and also may be involved in the acute morphine effects mediated via postsynaptic mechanisms in both placebo and morphine WD rats.

As PTX is an inhibitor of Gi/o, we expected to prevent the acute morphine-induced decrease in the amplitudes and/or frequencies of CeA mIPSCs. Surprisingly, PTX pre-incubation did not completely prevent acute morphine-induced inhibition or enhancement of CeA mIPSCs from placebo or morphine WD rats. In placebo rats, acute morphine decreased mean mIPSC frequencies or amplitude in 4 of 9 neurons, as determined by the Kolmogorov-Smirnoff test for individual cells (data not shown). Similarly, acute morphine application increased the amplitude or frequency of mIPSCs in an additional 4 cells. In WD rats, PTX also did not prevent the acute morphine reduction or enhancement of the mIPSCs: 2 of 6 neurons still showed increased mean frequencies (178.0 ± 29.4% of baseline, *n* = 2 from 2 rats) or 3 neurons showed decreases in both frequency (71.0% of baseline, *n* = 3 from 3 rats) and amplitude (82.2% of baseline) of the mIPSCs, respectively (data not shown). These findings suggest that in addition to a PTX-sensitive Gi/o pathway, other PTX-insensitive mechanisms may be involved in the inhibitory effects of acute morphine on GABAergic transmission in the CeA.

## Discussion

In this study, we assessed neuronal adaptations of the GABAergic and opioid systems in the CeA during naloxone-precipitated morphine withdrawal. We found increased CeA basal GABA release in morphine dependent rats during naloxone-precipitated withdrawal. Acute morphine application had mixed effects on the GABAergic transmission in the CeA neurons in this model of naloxone-induced morphine withdrawal. Our study also showed that a continuous exposure to naloxone during the naloxone-precipitated WD does not prevent acute morphine-induced modulation of GABAergic transmission. Under our experimental conditions, the acute morphine-induced inhibition of GABAergic transmission is very likely mediated by PTX-insensitive mechanisms in many CeA neurons, but we cannot rule out the possibility that a PTX-sensitive mechanism is involved in some CeA neurons as well. On the other hand, an activated cAMP signaling pathway is a likely molecular mechanism underlying the increase in basal GABAergic transmission and acute morphine-induced potentiation of GABAergic transmission in the CeA.

To model the naloxone-precipitated morphine withdrawal, we performed all recordings with naloxone and naloxone plus morphine co-application in the bath during recordings from the CeA neurons from the placebo and morphine WD rats, respectively. It has been shown that there is a tonic inhibition of GABAergic transmission mediated by MORs in the CeA. In addition, acute application of MOR antagonists prevents the effects of acute MOR agonists on GABAergic transmission (Zhu and Pan, 2004; Finnegan et al., 2005; Kang-Park et al., 2009; Bajo et al., 2011). Thus, our findings that acute morphine modulates GABAergic transmission in the CeA despite the presence of naloxone in the bath may suggest an incomplete inhibition of the CeA opioid system by naloxone or development of tolerance to naloxone during the naloxone-precipitated withdrawal. Similarly, previous studies have shown attenuation of naloxone-precipitated withdrawal symptoms by inhibition of enkephalinase activity (suggesting an increase in endogenous enkephalin levels; (Haffmans and Dzoljic, 1987; Haffmans et al., 1987) and induced heroin reinstatement during naloxone-precipitated WD by a priming injection of heroin (Shaham et al., 1996). Mechanisms underlying these paradoxical findings may involve both opioid-dependent and non-opioid-dependent (e.g., activation of glia via toll-like receptor 4) (Hutchinson et al., 2007, 2012; Watkins et al., 2009; Lewis et al., 2012) mechanisms. With regard to opioid dependent mechanisms, we hypothesize that a prolonged naloxone exposure (more than 1 h) could lead to a continuous blockade of the MOR-dependent tonic inhibition of GABAergic transmission and elicit tolerance and/or adaptation of the CeA opioid system. Changes in messaging pathways, especially in cAMP and PKA signaling, and perhaps Gz activation as well, may develop under the continuous presence of naloxone and thus mediate the acute morphine modulation of the GABAergic transmission.

The effects of naloxone on the opioid system are determined by a previous opioid treatment. Naloxone acts as a neutral agonist in MOR/DOR agonist-naïve cells cultures and as an inverse agonist after a previous MOR/DOR exposure (Wang et al., 2001). Thus, our findings of increased basal GABA release in some CeA neurons from the morphine WD rats compared to placebo rats may support the idea of naloxone acting as an inverse agonist in the morphine WD rats or as a neutral antagonist in the CeA in placebo rats. The increased GABA release in the CeA during naloxone-precipitated withdrawal is in accord with the enhanced GABAergic synaptic transmission found in other brain regions after such naloxone induced WD (Bonci and Williams, 1997; Chieng and Williams, 1998; Ingram et al., 1998; Jolas et al., 2000; Hack et al., 2003; Madhavan et al., 2010a,b). Our group (Bajo et al., 2008, 2009) and others (Madhavan et al., 2010a; Meye et al., 2012) reported that acute morphine increases GABAergic transmission during spontaneously-morphine WD rats and naloxone-precipitated withdrawal combined with recordings without naloxone showed similar inverse effects of acute MOR agonists on GABAergic transmission during morphine withdrawal. Overall, these findings indicate that MOR-induced potentiation of GABAergic transmission during morphine withdrawal is a general neuroadaptive mechanism associated with morphine withdrawal. However, a role for non-opioid mechanisms cannot be ruled out and require further investigation.

The molecular mechanism of both the action-potential independent increase in GABA release and the morphine-induced potentiation of GABAergic transmission in the CeA appears to be mediated by a hyperactivation of cAMP, because RP pretreatment decreased the mean frequencies of basal mIPSCs and prevented the acute morphine enhancement of GABAergic transmission. There are numerous studies reporting a compensatory increase in PGE_1_- or forskolin-stimulated adenylyl cyclase activity in neuronal cells chronically exposed to opioids upon removal of the opioids (Bailey and Connor, 2005; Williams et al., 2013). A superactivation of cAMP also has been reported to mediate DAMGO-induced enhancement of GABA transmission in the VTA (Madhavan et al., 2010a; Meye et al., 2012). In general, the excitatory effects occurring during opioid tolerance and dependence is thought to be mediated by an additive or synergistic effect of a loss of adenylyl cyclase inhibition by Gi/o and stimulation of adenylyl cyclases by both Gs and its associated Gβγ (Wang and Burns, 2006). Thus, we assume that both mechanisms, a switch in MOR G protein coupling from cAMP-inhibitory Gi/o to cAMP-stimulatory Gs (Crain and Shen, 1998; Wang et al., 2005) and a stimulation of adenylyl cyclase by Gβγ subunits, may mediate superactivation of cAMP during morphine withdrawal in the CeA. It has been shown that chronic morphine treatment maintains the association of Gs with MORs and diminishes the association of Gi/o with MORs (Chakrabarti et al., 2005; Wang and Burns, 2006). Studies on cell cultures also showed that chronic morphine-induced interaction of Gβγ with adenylyl cyclase can occur without alteration of the G protein coupling profile (Chakrabarti et al., 2001). These findings support the hypothesis that both of these events can contribute to enhanced activation of the cAMP following chronic morphine treatment (Wang et al., 2005; Wang and Burns, 2006). The chronic morphine treatment-induced reduction of MOR-Gi/o coupling, paralleled with increased Gs coupling and a direct interaction of Gβγ with adenylate cyclase, has been found in the striatum, periaqueductal gray, and spinal cord (Kitanaka et al., 2008).

MORs preferentially couple to the PTX-sensitive G proteins, Gi, and Go, to inhibit the adenylyl cyclase/cAMP pathway (Connor and Christie, 1999). We found that Gi/o plays a role in the action potential-independent GABA release in CeA, as PTX pre-treatment reduced the mean frequencies of mIPSCs in the CeA neurons from morphine WD rats but had no effects in placebo rats. This concurs with studies in cultured cell lines showing opioid-induced adenylyl cyclase superactivation that was sensitive to PTX treatment, implicating the involvement of PTX-sensitive Gi/o proteins in mediating these adaptive responses (Tso and Wong, 2000; Steiner et al., 2005). Another possibility may include a PTX-pretreatment induced substitution of Gi/o by Gz. Despite a similarity in inhibition of adenylyl cyclase and K^+^ channels, Gz exhibits unique biochemical, and regulatory properties (Ho and Wong, 2001) that may underlie the decrease in basal mIPSCs observed in the CeA of WD rats. Our results showing acute morphine-induced reduction of GABAergic transmission following PTX-pre-treatment of the CeA slices suggest that both PTX-sensitive as well as PTX-insensitive mechanisms, such as Gz or Gβγ subunits, may be involved in the inhibitory effects of acute morphine on GABAergic transmission in the CeA. MORs have been reported to modulate both PTX-sensitive Gi/oproteins as well as the PTX-insensitive Gz proteins (Sanchez-Blazquez et al., 2000). Gz also inhibits adenylyl cyclase (Sadana and Dessauer, 2009) and is thought to be involved in the regulation of ion channel activities and exocytosis (Ho and Wong, 2001). Morphine is a strong activator of Gz (Garzon et al., 1998), and the antinociceptive activity of morphine (Sanchez-Blazquez et al., 2001; Garzon et al., 2011) and analgesic desensitization are strongly decreased after Gz depletion (Garzon et al., 2009). As Gz is expressed in the amygdala (Carrasco et al., 2004), findings of alleviated morphine dependence and subsequent naloxone-precipitated withdrawal syndrome following intracerebroventricular administration of a Gz-specific antiserum (Sanchez-Blazquez and Garzon, 1994) suggest a potential involvement of Gz in opioid signaling in the CeA during morphine withdrawal.

The overall effects of chronic morphine exposure in a given CeA neuron may also depend on the presence of particular AC isoforms in those cells (Avidor-Reiss et al., 1997; Watts and Neve, 2005). Most cells express two or more AC isoforms and nearly all AC isoforms are expressed in the brain (Sadana and Dessauer, 2009). In general, a chronic treatment followed by agonist withdrawal has been shown to increase the activity of AC-I, AC-V, AC-VI, and AC-VIII isoforms, while reducing activity of the AC-II, IV, and VII isoforms (Sunahara and Taussig, 2002). In morphine dependence, an upregulation of AC signal transduction components is found in brain regions associated with drug reinforcement and withdrawal (Sadana and Dessauer, 2009), and is mediated by the AC-I, V, VI, and VIII isoforms (Avidor-Reiss et al., 1997; Chakrabarti et al., 2001). By contrast, an over-expression of ACVII results in enhanced acute and chronic actions of morphine, and leads to more rapid development of tolerance (Yoshimura et al., 2000).

In summary, these results indicate a cell-specific neuroadaptation to chronic morphine treatment of the opioid system and its effects on GABAergic transmission in CeA neurons. The cell-type specific responses to acute morphine application during chronic morphine treatment and morphine WD raise several questions: (1) What afferent projections and what CeA neuronal populations undergo neuroadaptive changes during the transition period leading to development of morphine dependence? (2) What mechanisms are regulating these chronic morphine-induced circuitry- and cell-specific neuroadaptative changes in the CeA? (3) What particular neuroadaptive changes of the opioid and neurotransmitter systems in the CeA play a critical role in development of morphine dependence and withdrawal? To address these issues, further studies will be required, focusing on characterization of cell types, circuitries, afferent projections and their targeted CeA neurons with regard to their neurotransmitter, neuropeptide, and cAMP contents.

### Conflict of interest statement

The authors declare that the research was conducted in the absence of any commercial or financial relationships that could be construed as a potential conflict of interest.
